# *BmFoxO* Gene Regulation of the Cell Cycle Induced by 20-Hydroxyecdysone in BmN-SWU1 Cells

**DOI:** 10.3390/insects11100700

**Published:** 2020-10-14

**Authors:** Qian Zhang, Jigui Yang, Peng Chen, Taihang Liu, Qin Xiao, Xiaolin Zhou, Ling Wang, Yanbi Long, Zhanqi Dong, Minhui Pan, Cheng Lu

**Affiliations:** 1 State Key Laboratory of Silkworm Genome Biology, Southwest University, Chongqing 400715, China; qianzhang126@126.com (Q.Z.); jiguiyang@126.com (J.Y.); pjchen@swu.edu.cn (P.C.); sitoyy@163.com (T.L.); XZ953179866@163.com (Q.X.); zhouxl126@126.com (X.Z.); 13243522501@163.com (L.W.); 15320206990@163.com (Y.L.); zqdong@swu.edu.cn (Z.D.); 2 Key Laboratory of Sericultural Biology and Genetic Breeding, Ministry of Agriculture and Rural Affairs, Southwest University, Chongqing 400715, China

**Keywords:** 20-Hydroxyecdysone, *BmFoxO*, *Bombyx mori*, cell cycle, developmental switching, EcR

## Abstract

**Simple Summary:**

Ecdysteroid titer determines the state of the cell cycle in silkworm (*Bombyx mori*) metamorphosis. However, the mechanism of this process is unclear. In this study, we reported that 20-Hydroxyecdysone (20E) can promote *BmFoxO* (*Bombyx mori* Forkhead box protein O) gene expression and induce *BmFoxO* nuclear translocation in BmN-SWU1 cells. Overexpression of the *BmFoxO* gene affects cell cycle progression, which results in cell cycle arrest in the G0/G1 phase as well as inhibition of DNA replication. Further investigations showed that the effect of 20E was attenuated after *BmFoxO* gene knockdown. The findings of this study confirmed that *BmFoxO* is a key mediator in the cell cycle regulation pathway induced by 20E. This suggests a novel pathway for ecdysteroid-induced cell cycle regulation in the process of silkworm metamorphosis, and it is likely to be conserved between Lepidoptera insects.

**Abstract:**

Ecdysteroid titer determines the state of the cell cycle in silkworm (*Bombyx* *mori*) metamorphosis. However, the mechanism of this process is unclear. In this study, we demonstrated that the *BmFoxO* gene participates in the regulation of the cell cycle induced by 20-Hydroxyecdysone (20E) in BmN-SWU1 cells. The 20E blocks the cell cycle in the G2/M phase through the ecdysone receptor (EcR) and inhibits DNA replication. The 20E can promote *BmFoxO* gene expression. Immunofluorescence and Western blot results indicated that 20E can induce *BmFoxO* nuclear translocation in BmN-SWU1 cells. Overexpression of the *BmFoxO* gene affects cell cycle progression, which results in cell cycle arrest in the G0/G1 phase as well as inhibition of DNA replication. Knockdown of the *BmFoxO* gene led to cell accumulation at the G2/M phase. The effect of 20E was attenuated after *BmFoxO* gene knockdown. These findings increase our understanding of the function of 20E in the regulation of the cell cycle in *B. mori*.

## 1. Introduction

During larval growth and metamorphosis, transition of the cell cycle status is necessary to determine the appropriate size and shape of the insect [1,2]. Developmental changes are controlled by the endocrine system, and many hormones interact to regulate insect growth and development [3]. Among these, the ecdysteroid hormone ecdysone, produced by the prothoracic gland (PG), regulates molting and metamorphosis in its active form [4,5,6]. The function of ecdysone has also been studied in relation to embryonic development [7], ovary development [8], and silk gland development [9]. The PG secretes ecdysone, which is converted to 20E that is active in the peripheral tissues including the fat body, midgut, and muscle [10]. Ecdysteroids generally function by activating the ecdysone receptor (EcR), a member of the transcription factor nuclear receptor family, to regulate the expression of specific genes [11].

A moderate increase in ecdysteroid titer can determine the developmental transition of the cell cycle status [12,13]. Nevertheless, the underlying molecular signaling pathways that combine ecdysteroid with cell cycle regulation are poorly understood. The only example of the signaling pathway was confirmed in the wing disc of *Drosophila melanogaster* [14,15]. In this case, 20E promoted the expression of the transcription factor *Crol* (Crooked legs) [15], which subsequently enhanced the expression of *dMyc* (the *Drosophila* ortholog of the proto-oncogene *c-Myc*), *Stg* (the *Drosophila* ortholog of *Cdc25*), and *Cyclin B* by inhibiting the Wg/Wnt pathway [16].

FoxO proteins are a subgroup of the forkhead transcription factor family [17]. FoxO proteins play an important regulatory role in many cellular processes, including the coordination of genes involved in rat denervated gastrocnemius muscle apoptosis [18], cellular differentiation in *Drosophila* [19], autophagy in tumor cells [20], and cell proliferation in glioblastoma [17]. FoxO is activated by 20E via upregulating PTEN (phosphatase and tensin homolog) expression to counteract insulin activity and promote proteolysis during *Helicoverpa armigera* molting [21]. However, the underlying molecular signaling pathways by which 20E and the *FoxO* gene regulate the cell cycle are unknown.

The present study aimed to illuminate the effects of the molecular pathway of 20E on cell cycle regulation in *Bombyx mori*. We verified the important role of the ecdysone receptor in the regulation of the cell cycle induced by 20E. We elucidated the potential molecular signaling pathways that combine 20E with *BmFoxO* gene regulation. We also analyzed the function of the *BmFoxO* gene in the regulation of the cell cycle of *Bombyx mori*. Overall, we demonstrated that the *BmFoxO* gene is an important regulator in 20E-induced cell cycle regulation.

## 2. Materials and Methods

### 2.1. Bioinformation Analysis

All of the homology sequences were searched from the National Center for Biotechnology Information (NCBI, http://www.ncbi.nlm.nih.gov/) and the silkworm genome database (SilkDB, https://silkdb.bioinfotoolkits.net/main/species-info/-1). The primers were designed by Primer Premier 5.0 software. The knockout sgRNA was designed by CRISPRdirect (http://crispr.dbcls.jp/).

### 2.2. Cell Culture and Transient Transfections

The cell line, BmN-SWU1, derived from silkworm ovaries, was cultured at 27 °C with TC-100 insect medium (United States Biological, Swampscott, MA, USA) supplemented with 10% fetal bovine serum (BI, Kibbutz Beit Haemek, Israel), 100 U/mL penicillin, and 100 μg/mL streptomycin (Gibco, Grand Island, NE, USA) [22]. Before transfection, pure plasmids were prepared using Plasmid Mini Kits (Qiagen, Hilden, Germany). Transfections with a mixture of plasmid and X-treme GENE HP DNA Transfection Reagent (Roche, Basel, Switzerland) were allowed to stand for 30 min, mixed in a 200 μL antibiotic-free and serum-free medium according to manufacturer instructions. After 6–8 h post transfection, the medium was replaced with normal medium.

### 2.3. Plasmid Construction

The *BmFoxO* cDNA was amplified with primers (forward 5′ GAAAGAAATCGCTTACAAAATCAG 3′ and reverse 5′ ATCTCCACAACTCATCACCCG 3′) and cloned into the pMD19-T vector (Takara, Dalian, China). The correct fragments were obtained by PCR using the primers (forward 5′ggggtaccATGTACCCATACGATGTTCCAGATTACGCTTCAATTCAGGAGGCGGCG3’ and reverse 5′gctctagaTCA AGCGTAATCTGGAACATCGTATGGGTAGTGGACCCAGGAGGGGGTGA3′) from pMD19-BmFoxO. The underlined sequences represent HA tag sequences. The PCR products and the insect expression vector pIZ/V5-His (Invitrogen, Carlsbad, CA, USA) were ligated using *Kpn*I and *Xba*I sticky ends to construct the final vector pIZ-BmFoxO. The PCR products were also connected to the vector pIZ-EGFP to fuse with the enhanced green fluorescent protein (GFP) gene to construct the final vector pIZ-BmFoxO-EGFP. The three AKT-phosphorylation sites in *BmFoxO* (T50A, S189A, S253A) were mutated by codon modification and gene synthesis (Genewiz, Suzhou, China) to construct the constitutively active/nuclear form of *BmFoxO* (BmFoxO-CA) [23]. The pIZ-BmFoxO-CA and pIZ-BmFoxO-CA-EGFP constructs were then generated with the same methods. Cas9-BmFoxO single guide RNA (sgRNA) recombinant plasmid (BmFoxO-KO) and Cas9-BmEcR sgRNA recombinant plasmid (EcR-KO) were constructed as previously described [24]. In our experiment, we analyzed mixed cultures including knockout and intact cells, and the percentage of knockout cells was around 40%.

### 2.4. 20E Treatment

First, 20E (Sigma Co., St. Louis, MO, USA) was dissolved in ethanol to make 20 mg/mL stock concentration. This was then diluted to 2 μg/μL working concentration using dimethyl sulfoxide (DMSO). The BmN-SWU1 cells were incubated in TC100 insect medium supplemented with 20E for a final concentration of 0.25 μg/mL. Control cells were treated with the same amount of DMSO.

### 2.5. Quantitative Real-Time Polymerase Chain Reaction (qRT-PCR)

Total RNA was purified from each sample using Total RNA Kit II (OMEGA, Norcross, GA, USA) and 1 μg of total RNA was reverse-transcribed into 20 μL of cDNA using PrimeScript RT Reagent Kit (Takara) according to manufacturer’s instructions. Primers (TsingKe, Chongqing, China) used for qRT-PCR were *BmFoxO*: forward 5′ AGCAGTTTCCAGTTGTCGCC 3′ and reverse 5′ GTCCGCTTGTGAGAAGTCTGTATT3′. The housekeeping gene, ribosomal protein gene (*rpl3*) (forward 5′ CGGTGTTGTTGGATACATTGAG 3′ and reverse 5′ GCTCATCCTGCCATTTCTTACT 3′), was used as the reference gene. QRT-PCR was carried out in 15 μL reaction volumes containing 1 μL of 5-fold diluted cDNA, 0.5 mM of each primer, and iTaq Universal SYBR Green Supermix (Bio-Rad, Hercules, CA, USA) in 96-well plates. The reaction conditions were 94 °C for 30 s, followed by 40 cycles at 95 °C for 5 s and 60 °C for 15 s. Then, the melt curve analysis was performed from 65 °C to 95 °C with a 0.5 °C increment for 5 s in each step.

### 2.6. Flow Cytometry

The cells were washed twice with PBS and fixed overnight with 75% ethanol. Then, the cells were washed with PBS and incubated with RNase A and propidium iodide (PI) for 30 min at 37 °C. The cells were then analyzed by CytoFLEX flow cytometer (Beckman Coulter, Brea, CA, USA).

### 2.7. BrdU Incorporation and Immunofluorescence

The cells were spiked with BrdU (Roche) at 1:200 for 2 h in TC-100 insect medium. Then, the cells were fixed in 4% paraformaldehyde for 15 min and washed three times with phosphate-buffered saline containing 5% Tween-20 (PBST, Beyotime, Shanghai, China). Then, the cells were blocked with 3% bovine serum albumin and 10% sheep serum in PBS (blocking solution) at 37 °C for 1 h. The cells were further incubated with anti-BrdU antibody (1:200; Roche) and anti-HA antibody (1:200; Abcam, Cambridgeshire, UK) in blocking solution for 1.5 h at 37 °C. Then, they were washed six times with PBST for 6 min each time and then incubated for 1 h with Alexa Fluor 555-conjugated donkey anti-rabbit IgG secondary antibody (1:500; Life Technologies, Rockville, MD, USA) and Alexa Fluor 488-conjugated donkey anti-mouse IgG secondary antibody (1:500; Life Technologies) in blocking solution. The cells were observed under a confocal microscope (Olympus, Tokyo, Japan).

### 2.8. MTT Assay

The MTT (3-[4,5-dimethylthiazol-2-yl]-2,5 diphenyl tetrazolium bromide) assay was used to determine cell proliferation ability. The transfected cells were harvested at different time points and counted. These cells were seeded into 96-well plates and 100 μL of the MTT solution (5 mg/mL) was added to each well. They were then incubated at 37 °C for 4 h. Cellular viability was determined at a wavelength of 570 nm.

### 2.9. Nuclear and Cytoplasmic Protein Extraction and Western Blot

The 2 × 10^5^ BmN-SWU1 cells were collected 48 h after transfection. After three washes in PBS, cells were collected by centrifugation at 1000× *g*. Nuclear and cytoplasmic fractionation was carried out using the Nuclear and Cytoplasmic Protein Extraction Kit following the manufacturer’s instructions (Beyotime, Shanghai, China). The BmN-SWU1 cells (2 × 10^5^) were plated in 6-well plates and transfected with 2 μg plasmid. At the indicated time points, cells were harvested for Western blotting. The cell samples were lysed using cell lysis buffer for Western and IP (Beyotime). The total protein concentration was determined using a BCA Protein Assay Kit (Beyotime). After SDS-PAGE, the proteins were transferred onto a hydrophilic polyvinylidene fluoride (PVDF) membrane (Roche) and incubated with indicated primary antibodies. Then, the membrane was further incubated with HRP-labeled secondary antibodies (Beyotime). The blots were visualized using a Clarity Western ECL Substrate (Bio-Rad).

### 2.10. Statistical Analysis

Results from three independent experiments are presented as means ± SD. Data were analyzed using the Student’s *t* test for comparison of two groups or two-way ANOVA for multiple groups (GraphPad Prism 6 Software). The number of asterisks represents the degree of significance with respect to *p* value. *p* values were provided as * *p* < 0.05; ** *p* < 0.01; *** *p* < 0.001.

## 3. Results

### 3.1. Regulation of the Cell Cycle by 20E

**Figure 1:**
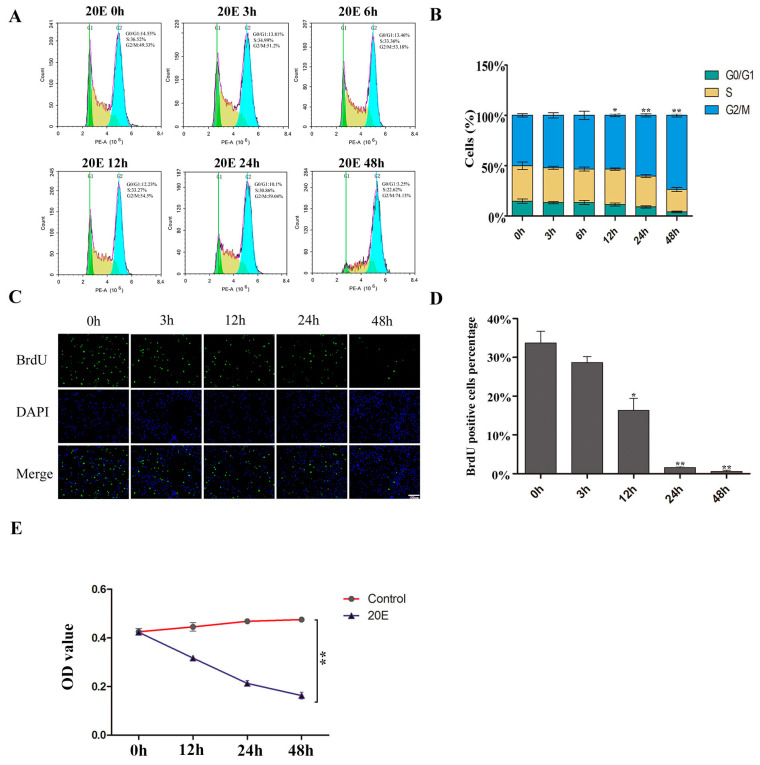
20-Hydroxyecdysone (20E) suppresses cell proliferation and induces cell cycle arrest. (**A**) The cell cycle of BmN-SWU1 cells after 0.25 μg/mL 20E induction at 0 h, 3 h, 6 h, 12 h, 24 h, and 48 h was analyzed by flow cytometry. (**B**) Statistical analysis of the cell percentage of each phase, including G0/G1, S, and G2/M (* *p* < 0.05, ** *p* < 0.01). (**C**) Anti-5-bromodeoxyuridine (anti-BrdU)-labeled BmN-SWU1 cells after 0.25 μg/mL 20E induction at 0 h, 3 h, 12 h, 24 h, and 48 h. Nuclei were stained with DAPI (4′,6-diamidino-2-phenylindole). Scale bars, 100 μm. (**D**) The ratio of positive cells labeled with BrdU (* *p* < 0.05, ** *p* < 0.01). (**E**) The MTT (3-[4,5-dimethylthiazol-2-yl]-2,5 diphenyl tetrazolium bromide) assay was used to construct the growth curve of BmN-SWU1 cells after 0.25 μg/mL 20E induction at 0 h, 12 h, 24 h, and 48 h (* *p* < 0.05, ** *p* < 0.01).

We added 20E to BmN-SWU1 cells at different time points to study cell cycle and cell proliferation activity. We measured the cell cycles in different time points by flow cytometry and found that the cells were gradually blocked to the G2/M phase. The effect occurred in a time-dependent manner. The G2/M phase cells increased from 49.33 ± 3.51% to 74.13 ± 2.11% at 48 h. At 48 h, the percentage of the cells in S phase showed a decrease (Figure 1A,B). We examined the effect of 20E on DNA replication at different time points. After treatment with 20E, the percentage of 5-bromodeoxyuridine (BrdU) positive cells gradually decreased. The percentage of BrdU-positive cells decreased from 34 ± 3.06% to 16 ± 3.05% at 12 h. Positive cells were 1.6 ± 0.31% at 24 h and 0.58 ± 0.35% at 48 h. All of the decreases were statistically significant (Figure 1C,D). MTT assay was used to generate a growth curve and DMSO was added, at different time points, as a control. The proliferation activity of BmN-SWU1 cells greatly decreased after the addition of 20E (Figure 1E). Together, these data indicate that 20E blocks the cell cycle in the G2/M phase and inhibits DNA replication.

### 3.2. 20E Can Regulate the Cell Cycle through BmEcR in BmN-SWU1 Cells

**Figure 2:**
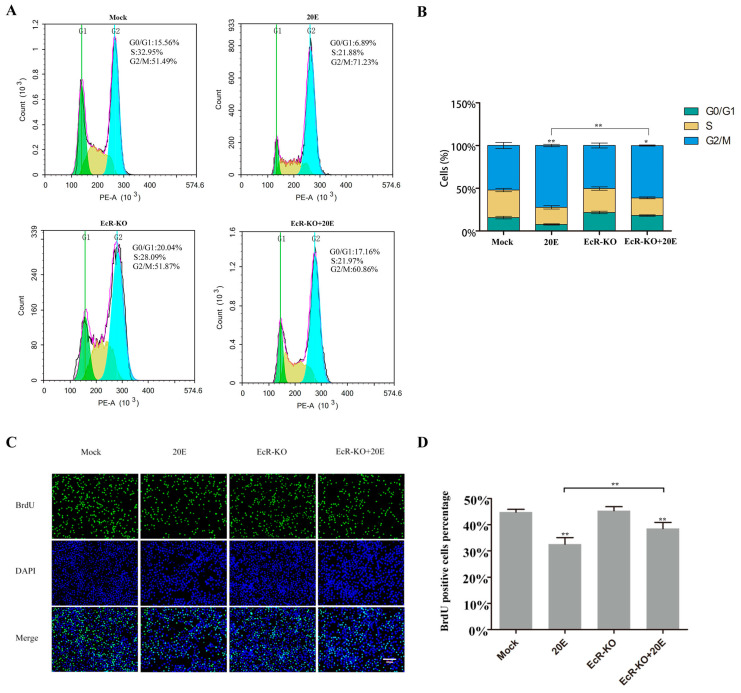
20E can regulate the cell cycle through BmEcR in BmN-SWU1 cells. (**A**) Cell cycle analysis of BmN-SWU1 cells incubated with 0.25 μg/mL 20E for 24 h after transfection with Cas9-BmEcR sgRNA recombinant plasmid (EcR-KO) or Cas9-Mock plasmid. (**B**) Statistical analysis of the cell percentage of each phase, including G0/G1, S, and G2/M phases (* *p* < 0.05, ** *p* < 0.01). (**C**) Anti-5-bromodeoxyuridine (anti-BrdU)-labeled BmN-SWU1 cells incubated with 0.25 μg/mL 20E for 24 h after transfection with Cas9-BmEcR sgRNA recombinant plasmid (EcR-KO) or Cas9-Mock plasmid. Nuclei were stained with DAPI. Scale bars, 100 μm. (**D**) Ratio of positive cells labeled with BrdU (* *p* < 0.05, ** *p* < 0.01).

To determine if the ecdysone receptor participated in the cell cycle regulation process by 20E, we used CRISPR/Cas9 technology to knockdown *BmEcR*. After 20E addition, the percentage of cells in the G2/M phase was significantly increased (71.23 ± 1.43%) compared to that in the control group (51.49 ± 3.55%). We added 20E after *BmEcR* knockdown and found that the percentage of the G2/M phase decreased significantly from 71.23 ± 1.43% to 60.86 ± 0.65% (Figure 2A,B). These results indicate that BmEcR is required for cell cycle regulation by 20E. We also measured the DNA replication by BrdU assay. Adding 20E reduced the percentage of BrdU-positive cells (from 44.4 ± 2.3% to 31 ± 2.50%). We added 20E after *BmEcR* knockdown and found that the percentage of BrdU-positive cells increased to 35 ± 2.34%, which alleviated the inhibitory effect of 20E (Figure 2C,D). These data demonstrate that 20E can inhibit the cell cycle progression in BmN-SWU1 cells.

### 3.3. The BmFoxO Gene Is Necessary for Cell Cycle Regulation Induced by 20E

**Figure 3:**
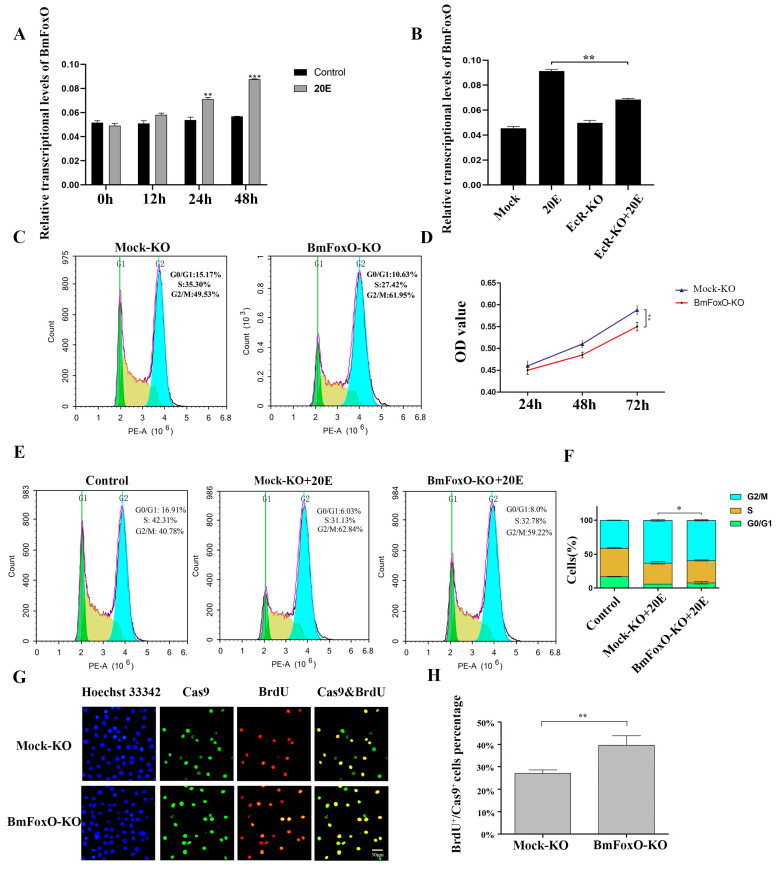
*BmFoxO* gene deficiency impairs cell cycle regulation by 20E. (**A**) The transcriptional levels of *BmFoxO* gene after 0.25 μg/mL 20E induction at 0 h, 12 h, 24 h, and 48 h were analyzed by qRT-PCR (* *p* < 0.05, ** *p* < 0.01, *** *p* < 0.001). (**B**) The transcriptional levels of *BmFoxO* gene in BmN-SWU1 cells incubated with 0.25 μg/mL 20E for 24 h after transfection with Cas9-BmEcR sgRNA recombinant plasmid (EcR-KO) or Cas9-Mock plasmid were analyzed by qRT-PCR (* *p* < 0.05, ** *p* < 0.01). (**C**) Cell cycle analysis of BmN-SWU1 cells transfected with Cas9-Mock plasmid (Mock-KO) or *BmFoxO* gene knockout plasmid (BmFoxO-KO), as determined by flow cytometry analysis. (**D**) Cell proliferation ability of the BmN-SWU1 cells transfected with Cas9-Mock plasmid (Mock-KO) or *BmFoxO* gene knockout plasmid (BmFoxO-KO) was detected by MTT assay at 24 h, 48 h, and 72 h (* *p* < 0.05, ** *p* < 0.01). (**E**) Cell cycle analysis of BmN-SWU1 cells incubated with 0.25 μg/mL 20E for 24 h after being transfected with Cas9-Mock plasmid (Mock-KO) or *BmFoxO* gene knockout plasmid (BmFoxO-KO) by flow cytometry. (**F**). Statistical analysis of the cell percentage of each phase, including G0/G1, S, and G2/M phases. Asterisk represents the significant difference between G2/M phase. (* *p* < 0.05, ** *p* < 0.01). (**G**). Anti-5-bromodeoxyuridine (anti-BrdU)-labeled BmN-SWU1 cells transfected with Cas9-Mock plasmid (Mock-KO) or *BmFoxO* gene knockout plasmid (BmFoxO-KO). Nuclei were stained with Hoechst 33342. (**H**). Ratio of Cas9 positive cells labeled with BrdU (* *p* < 0.05, ** *p* < 0.01). Scale bars, 50 μm.

It has been reported that 20E inhibits FoxO phosphorylation and results in its nuclear translocation [21]. Activated FoxO promotes proteolysis during larval *H. armigera* molting [21]. We cloned the *BmFoxO* gene from larval cDNA of the *B. mori Dazao* strain, and this was transfected into BmN-SWU1 cells. To determine whether 20E could induce *BmFoxO* nuclear translocation, we incubated BmFoxO-overexpressed BmN-SWU1 cells with 20E. Six hours later, we analyzed the subcellular localization of *BmFoxO* by immunofluorescence. In the DMSO control group, *BmFoxO* was mainly distributed in the cytoplasm. However, after 6 h of incubation with 20E, *BmFoxO* had significantly increased nuclear localization (Supplementary Materials Figure S1A). Furthermore, we counted the proportion of BmFoxO-positive cells with nuclear localization and found that such cells accounted for almost 50% of the total (Figure S1B). Western blotting analysis confirmed that *BmFoxO* protein accumulated in the nuclei of the BmN-SWU1 cells after 20E administration (Figure S1C,D). These results revealed that 20E induces *BmFoxO* nuclear translocation. Given the finding that 20E promotes *BmFoxO* nuclear translocation, we suggest that the *BmFoxO* gene plays an important role in the cell cycle regulation pathway by 20E. Next, we tested the transcriptional levels of *BmFoxO* gene after adding 20E and found that 20E can significantly increase the transcriptional levels of *BmFoxO* (Figure 3A). To validate the role of the *BmFoxO* gene by 20E regulation, we used CRISPR/Cas9 technology to knockdown the *BmEcR* gene (Figure S2B). After *BmEcR* gene knockdown, the effect of 20E administration was weakened and the transcriptional levels of *BmFoxO* gene were significantly decreased (Figure 3B).

To further investigate whether *BmFoxO* gene is necessary for the regulation of the cell cycle by 20E, we constructed a *BmFoxO* gene knockout vector using CRISPR/Cas9 technology and then detected cell cycle and cell proliferation activity. Cas9-BmFoxO single guide RNA (sgRNA) recombinant plasmid (BmFoxO-KO) and Cas9-mock sgRNA recombinant control plasmid were separately transfected into BmN-SWU1 cells. We collected the cells at 72 h post transfection and performed flow cytometry analysis to determine the cell cycle distribution. *BmFoxO* gene deficiency increased the percentage of cells in the G2/M stage to 61.95 ± 1.3% compared with 49.53 ± 1.6% in the control. These results showed that *BmFoxO* gene deficiency led to cell accumulation at the G2/M phase (Figure 3C). We analyzed whether the *BmFoxO* gene is necessary for the proliferation of BmN-SWU1 cells. MTT assay results showed that *BmFoxO* gene deficiency inhibits the proliferation of BmN-SWU1 cells (Figure 3D). We added 20E to BmN-SWU1 cells for 24 h and the percentage of cells in G2/M phase increased significantly (62.84 ± 1.3%) relative to the control (40.78 ± 0.2%), but the effect of 20E was attenuated after *BmFoxO* gene knockdown (59.22 ± 0.9%) (Figure 3E,F). BrdU assay also revealed that *BmFoxO* gene deficiency induced a higher percentage of BrdU-positive cells, with 39 ± 8.6% compared to 27 ± 2.9% of the control (Figure 3G,H). These results indicate that *BmFoxO* gene deficiency alleviates the proportion of cells arrested in the G2/M phase by 20E, suggesting that the *BmFoxO* gene is a crucial regulator in the 20E-induced cell cycle regulation pathway.

### 3.4. BmFoxO Inhibits Cell Proliferation and Causes Cell Cycle Arrest

**Figure 4:**
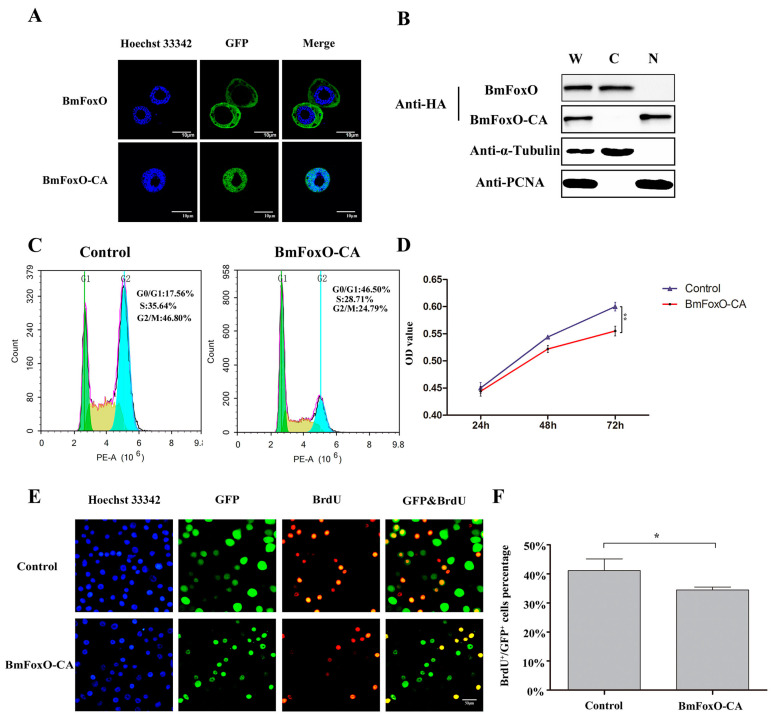
*BmFoxO* gene regulates DNA replication and cell cycle progression. (**A**) BmN-SWU1 cells were transfected with pIZ-BmFoxO-EGFP plasmids or pIZ-BmFoxO-CA-EGFP plasmids for 48 h. Immunofluorescence analysis of the localization of *BmFoxO* and BmFoxO-CA, separately. Nuclei were stained with Hoechst 33342. Scale bars, 10 μm. (**B**) BmN-SWU1 cells were transfected with pIZ-BmFoxO-EGFP plasmids or pIZ-BmFoxO-CA-EGFP plasmids for 72 h. Western blot analysis of the localization of *BmFoxO* and BmFoxO-CA, separately. W represents whole cell lysates, C indicates the cytoplasmic proteins, and N shows the nuclear proteins. (**C**) Cell cycle analysis of BmN-SWU1 cells transfected with control plasmids or pIZ-BmFoxO-CA-EGFP plasmids by flow cytometry. (**D**) The cell proliferation ability of BmN-SWU1 cells transfected with control plasmids or pIZ-BmFoxO-CA-EGFP plasmids was detected by MTT assay at 24 h, 48 h, and 72 h (* *p* < 0.05, ** *p* < 0.01). (**E**) Anti-5-bromodeoxyuridine (anti-BrdU)-labeled BmN-SWU1 cells transfected with control plasmids or pIZ-BmFoxO-CA-EGFP plasmids. Green fluorescence represents positive transfected cells. Nuclei were stained with Hoechst 33342. Scale bars, 50 μm. (**F**) Ratio of GFP positive cells labeled with BrdU (* *p* < 0.05, ** *p* < 0.01).

There are three phosphorylation sites (T50, S189, S253) in the amino acid sequence of the *BmFoxO* gene. All of the sites occur within the AKT consensus target sequence, RXRXX(S/T) [23]. To directly evaluate the function of the *BmFoxO* gene, we constructed overexpression vectors for normal *BmFoxO* as well as the constitutively active/nuclear form of *BmFoxO* (BmFoxO-CA). Subcellular localization of *BmFoxO* and BmFoxO-CA was detected by immunofluorescence. *BmFoxO* tagged with green fluorescent protein (GFP) was distributed in the cytoplasm, whereas BmFoxO-CA tagged with GFP was exclusively localized in the nucleus (Figure 4A). Western blot results confirmed that *BmFoxO* was distributed in the cytoplasm, whereas BmFoxO-CA was primarily localized in the nucleus (Figure 4B). Therefore, we used BmFoxO-CA for subsequent experiments.

To investigate whether the *BmFoxO* gene is involved in the regulation of cell cycle progression, we performed flow cytometry analysis for the BmFoxO-CA overexpressed BmN-SWU1 cells at 72 h post transfection. Surprisingly, the percentage of G0/G1 phase cells in the BmFoxO-CA overexpression groups directly increased to 46.50 ± 2.1%, in contrast to 17.56 ± 1.5% in the control groups (Figure 4C). The MTT assay revealed that BmFoxO-CA overexpression inhibited the proliferation activity of BmN-SWU1 cells (Figure 4D). To further investigate whether *BmFoxO* overexpression affects DNA replication, we directly labeled the BmFoxO-CA transfected BmN-SWU1 cells with 5-bromodeoxyuridine (BrdU). After BmFoxO-CA overexpression, there was a reduction in the percentage of BrdU-positive cells (34 ± 1.0%) compared to that in the control (41% ± 4.0), indicating that the relative rate of DNA synthesis in BmFoxO-CA overexpressed cells was reduced (Figure 4E,F). These results strengthen the conclusion that the *BmFoxO* gene has a key role in cell proliferation inhibition as well as in cell cycle arrest.

## 4. Discussion

The variety of cell cycle responses to the different 20E concentrations suggests a possible mechanism for developmental switching [25]. The 20E triggers transcriptional changes that regulate the developmental processes of the cell cycle in *D. melanogaster* [16]. In the present study, we found that 20E plays a crucial role in the cell cycle regulation process. The 20E led to cell cycle arrest in the G2/M cell phase through the EcR receptor while inhibiting DNA replication. In general, 20E functions by activating the ecdysone receptor (EcR) to regulate the expression of specific genes [11]. Flow cytometry and the BrdU assay demonstrated that knocking down the receptor EcR can eliminate the effect of 20E.

We also found that 20E can upregulate the transcription level of the *BmFoxO* gene. Immunofluorescence and Western blot results indicated that 20E regulated *BmFoxO* nuclear translocation in BmN-SWU1 cells. The distribution of FoxO depends on whether it has been phosphorylated. Non-phosphorylated FoxO can enter the nucleus to regulate downstream target genes [26]. In other lepidopteran insects, such as *H. armigera*, it has been reported that 20E can directly upregulate the expression of PTEN and FoxO through ecdysone receptor B1 (EcRB1) and the ultraspiracle protein (USP1). PTEN inhibits the phosphorylation of AKT, thereby repressing FoxO phosphorylation, resulting in FoxO nuclear translocation [21]. However, once it has been phosphorylated, FoxO is inactive, which results in its nuclear export and cytoplasmic retention as well as the inhibition of target gene expression [27]. Moreover, in *H. armigera*, insulin induces FoxO phosphorylation and cytoplasmic localization via AKT [28]. In mammals, *FoxO* is maintained in the cytoplasm under insulin regulation after phosphorylation by the phosphorylated protein kinase B (PKB) [29].

When we added 20E to BmN-SWU1 cells for 24 h, the percentage of cells in the G2/M phase significantly increased relative to the control. However, the effect of 20E was attenuated after *BmFoxO* gene knockdown. Based on these results, it can be concluded that the *BmFoxO* gene plays a special role in the cell cycle regulation pathway induced by 20E. *BmFoxO* is a transcription factor; it must enter the nucleus to perform its function. Thus, we constructed overexpression vectors for BmFoxO-CA, which is the constitutively active/nuclear form of *BmFoxO*. Unexpectedly, without adding 20E, the cell cycle was blocked in the G0/G1 phase after overexpression of BmFoxO-CA. *BmFoxO* relies on downstream target genes to regulate the cell cycle. We speculate that 20E may directly act on the target genes of *BmFoxO*, thereby shaping the functional diversity of *BmFoxO* in cell cycle regulation. It is conceivable that 20E not only drives *BmFoxO* to regulate cell cycle related genes but also uses other regulatory mechanisms to determine the final stage of the cell cycle.

In human cells, the target genes of FoxO protein induced in cell cycle regulation include cyclin dependent kinase inhibitor 1B (*KIP1*, also named as *p27*), growth arrest and DNA damage inducible (*GADD45*), and DNA damage binding protein 1 (*DDB1*) [30,31]. Determining the target genes of *BmFoxO* involved in cell cycle regulation will be a goal of future research.

In summary, this is the first study to report the physiological role of the *BmFoxO* gene as a key mediator in the 20E-induced cell cycle regulation pathway. This suggests a novel pathway for ecdysteroid-induced cell cycle regulation in the process of silkworm metamorphosis, and it is likely to be conserved between Lepidoptera insects.

## Supplementary Materials

The following are available online at https://www.mdpi.com/2075-4450/11/10/700/s1, Figure S1. 20E regulates *BmFoxO* nuclear translocation in BmN-SWU1 cells. (A) *BmFoxO* translocates to the nucleus after 20E induction in TC100 medium with 10% FBS. Cells were incubated with 0.25 μg/mL 20E for 6 h. Cells incubated with the same amount of DMSO for 6 h were treated as a control. Red fluorescence indicates the *BmFoxO* protein with HA tag. Blue, nuclei (Hoechst 33342). Scale bars, 10 μm. (B) Statistical analysis of the percentage of *BmFoxO* positive cells with nuclear distribution in A (* *p* < 0.05, ** *p* < 0.01). (C) After 48 h of transfection with the pIZ-*BmFoxO* plasmids, BmN-SWU1 cells were incubated with 0.25 μg/mL 20E or the same amount of DMSO for another 6 h. Then, the cytoplasmic and nuclear proteins were separated and detected by Western blotting. W represents whole cell lysates, C indicates the cytoplasmic proteins, and N shows the nuclear proteins. *BmFoxO* protein fused with HA tag was detected by HA antibody. In addition, α-tubulin was employed as the internal reference for cytoplasmic proteins and PCNA was used as the internal reference for nuclear proteins. (D) Statistical analysis of the relative proportion of nucleus distributed *BmFoxO* proteins after 0.25 μg/mL 20E incubation for 6 h (* *p* < 0.05, ** *p* < 0.01). Figure S2. (A) CRISPR/Cas9 vector construction. (B) DNA sequencing analysis of the CRISPR/Cas9 target sites in *BmFoxO* and *BmEcR* genes. The *BmFoxO* and BmEcR sequences of WT are shown at the top. The target sequence of sgRNA is indicated in blue. Deletions are indicated by dashes. The mutated bases are shown in red. (C) The nucleotide sequence (NM_001202535) and deduced amino acid sequence (NP_001189464.1) of the *BmFoxO* gene. The Forkhead domain is shown in yellow background. The FOXO-TAD domain is shown in cyan background.
