# Supplementary material for: BmFoxO Gene Regulation of the Cell Cycle Induced by 20-Hydroxyecdysone in BmN-SWU1 Cells
Source: Insects. 2020 Oct 14;11(10):700. doi: 10.3390/insects11100700 (PMC7602224; doi:10.3390/insects11100700)
Supplement: Supplementary file 1 [file insects-11-00700-s001.pdf]

Supplementary Materials

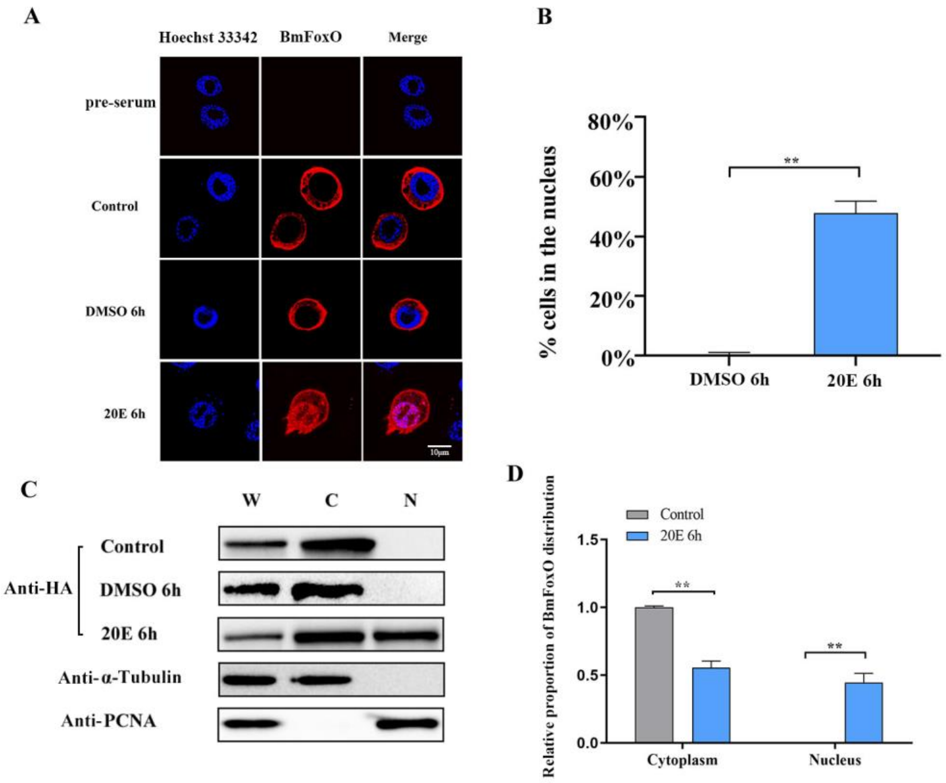

Figure S1. 20E regulates BmFoxO nuclear translocation in BmN-SWU1 cells. (A) BmFoxO translocates to the nucleus after 20E induction in TC100 medium with 10% FBS. Cells were incubated with 0.25 μg/mL 20E for 6 h. Cells incubated with the same amount of DMSO for 6 h were treated as a control. Red fluorescence indicates the BmFoxO protein with HA tag. Blue, nuclei (Hoechst 33342). Scale bars, 10 μm. (B) Statistical analysis of the percentage of BmFoxO positive cells with nuclear distribution in A (* $p < 0.05$, ** $p < 0.01$). (C) After 48 h of transfection with the pIZ-BmFoxO plasmids, BmN-SWU1 cells were incubated with 0.25 μg/mL 20E or the same amount of DMSO for another 6 h. Then, the cytoplasmic and nuclear proteins were separated and detected by Western blotting. W represents whole cell lysates, C indicates the cytoplasmic proteins, and N shows the nuclear proteins. BmFoxO protein fused with HA tag was detected by HA antibody. In addition, α-tubulin was employed as the internal reference for cytoplasmic proteins and PCNA was used as the internal reference for nuclear proteins. (D) Statistical analysis of the relative proportion of nucleus distributed BmFoxO proteins after 0.25 μg/mL 20E incubation for 6 h (* $p < 0.05$, ** $p < 0.01$).

A

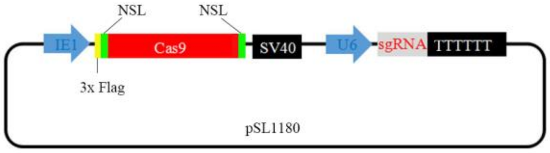

B

BmFoxO	gRNA	PAM	*BmEcR*	gRNA	PAM
TGCCAACG	ACCTCGTCATCTACTTCAGG	CGG	AATGTGATC	CACCTCCGCCCGAGGCCGCC	AGG
TGCCAACG	ACCTCGTCATCTAC----GG	CGG	AATGTGATC	CA---------------GCC	AGG
TGCCAACG	ACCTCGTCATCTAC-----G	CGG	AATGTGATC	CA-----GCC----------	AGG
TGCCAACG	----------CTACTTCAGG	CGG	AATGTGATC	CA------------GCC---	AGG
TGCCAACG	GCCTCGT----------AGG	CGG	AATGTGATC	CACCTCC---------CGCC	AGG
TGCCAACG	ACCTCGTCATCTACT-----	CGG	AATGTGATC	CACCTCCGCCC---TCCGCC	AGG

C

```
   1ATGTCAATTCAGGGAGGCGGCGGTTACCAGAGCCCTTGGTCATCTCAGACGGGGCTGTCAGAACTAGAGGGAACAATGGCAGAGTTGGAA
   1 M  S  I  Q  G  G  G  G  Y  Q  S  P  W  S  S  Q  T  G  L  S  E  L  E  G  T  M  A  E  L  E
  91CCATTAGGTGAACTGGCTGAGGTTGGCTTCGAGCCACAGACGCGCGCGCGATCCAACACGTGGCCGCTTCCAAGGCCAGACAACTACGTC
  31 P  L  G  E  L  A  E  V  G  F  E  P  Q  T  R  A  R  S  N  T  W  P  L  P  R  P  D  N  Y  V
 181GAGCAGGTAGACGAAGCCGGCTCTAAGAAGAATTCCAACCAGAATCTATCTGGAGCCCCACCAATACCAGCGAAGAAAAATTCATCGCGT
  61 E  Q  V  D  E  A  G  S  K  K  N  S  N  Q  N  L  S  G  A  P  P  I  P  A  K  K  N  S  S  R
 271CGCAACGCCTGGGGCAATCTTTCGTACGCAGATCTCATAACGCAAGCCATCACCTCGGCACAGGACAACAGGCTCACACTGTCGCAGATA
  91 R  N  A  W  G  N  L  S  Y  A  D  L  I  T  Q  A  I  T  S  A  Q  D  N  R  L  T  L  S  Q  I
 361TACGAGTGGATGGTCCAGAATGTGCCATATTTCAAAGACAAAGGGGATAACAATTCATCCGCAGGATGGAAGAATTCGATAAGACACAAC
 121 Y  E  W  M  V  Q  N  V  P  Y  F  K  D  K  G  D  N  N  S  S  A  G  W  K  N  S  I  R  H  N
 451CTGTCTCTTCACAACCGCTTCATGAGAGTTCAGAATGAGGGTACTGGCAAGTCATCATGGTGGATGATCAATCCCGACGCCAAGCCCGGA
 151 L  S  L  H  N  R  F  M  R  V  Q  N  E  G  T  G  K  S  S  W  W  M  I  N  P  D  A  K  P  G
 541AAATCCGTCAGGAGGCGAGCCGCTTCGATGGAAACCTCGAAATTCGAGAAACGAAGAGGAAGGGTAAAGAAAAAGACCGAAGCTTTAAGA
 181 K  S  V  R  R  R  A  A  S  M  E  T  S  K  F  E  K  R  R  G  R  V  K  K  K  T  E  A  L  R
 631AATGGAGCCACAGCTGATGCAACACCTAGCCCAAGTAGCTCAGTATCGGAGAGCATCGACATATTCACAGACTCACCAATGCACAGCAGT
 211 N  G  A  T  A  D  A  T  P  S  P  S  S  S  V  S  E  S  I  D  I  F  T  D  S  P  M  H  S  S
 721TTCCAGTTGTCGCCCGACTTCCGCCAGCGTGCTCCATCAAACGCTTCATCTTGCGGACGTCTATCTCCAATTCCTTCAATGATCCCTTCA
 241 F  Q  L  S  P  D  F  R  Q  R  A  P  S  N  A  S  S  C  G  R  L  S  P  I  P  S  M  I  P  S
 811GAGCCAGATTGGAAGCAAGAATACGCGAATGACTACGCCACCAATACAGACTTCTCACAAGCGGACTACACGCAAGAGGAACAGTTGGCA
 271 E  P  D  W  K  Q  E  Y  A  N  D  Y  A  T  N  T  D  F  S  Q  A  D  Y  T  Q  E  E  Q  L  A
 901GGTTCGCTGGCCGATTCCATGAAGCTGCAGGGAGCCGACCCATTCCTGAACCCGTACGTACCAACGACCTCGTCATCTACTTCAGGCGGT
 301 G  S  L  A  D  S  M  K  L  Q  G  A  D  P  F  L  N  P  Y  V  P  T  T  S  S  S  T  S  G  G
 991AGTTTTCGGTATGGATCCTACGGAACCTGCCCAAGACACCCTCACGGAGGCTGCACGTGTGCCTCTATGTACTCGCATCCTGCCCATCCT
 331 S  F  R  Y  G  S  Y  G  T  C  P  R  H  P  H  G  G  C  T  C  A  S  M  Y  S  H  P  A  H  P
1081GCCCACCCCACACACCCACATCAGCATGCCTTGGATCATTTCGTCAGGCCACCGCCGTCTGCTGATCAAGCCGATATCATGCAAACGGGT
 361 A  H  P  T  H  P  H  Q  H  A  L  D  H  F  V  R  P  P  P  S  A  D  Q  A  D  I  M  Q  T  G
1171AATAGGCAAACTCAAATGGTGACGACGTCGGATCCCGCTTTGATGAATGGTGGAATGATGGTGCAGGCTGGTGGAATGGGTCCAACGACT
 391 N  R  Q  T  Q  M  V  T  T  S  D  P  A  L  M  N  G  G  M  M  V  Q  A  G  G  M  G  P  T  T
1261GTCATGGGACAGATAATGGGCGCCTTGAACTCTGGTTTGGTAGAGGACTTAAATTTCGAAGCATTGGAACATGGCTTCGACTGCAATGTA
 421 V  M  G  Q  I  M  G  A  L  N  S  G  L  V  E  D  L  N  F  E  A  L  E  H  G  F  D  C  N  V
1351GATGAGGTCATAAAACACGAACTCAGCATGGACGGGTCCTTAGACTTCAATTTCCCTCAGCAGCAGACCGCAATGGCTGCCGAAGCAGAG
 451 D  E  V  I  K  H  E  L  S  M  D  G  S  L  D  F  N  F  P  Q  Q  Q  T  A  M  A  A  E  A  E
1441AGCCAGTTCGTTGCGCCCGCACCTCCTGTACCCACCACGCTGTCCGGGGGCGGAACCCCCCGCACCGCCTACTCCGTCACCCCCTCCTGG
 481 S  Q  F  V  A  P  A  P  P  V  P  T  T  L  S  G  G  G  T  P  R  T  A  Y  S  V  T  P  S  W
1531GTCCACTGA
 511 V  H
```

Figure S2. (A) CRISPR/Cas9 vector construction. (B) DNA sequencing analysis of the CRISPR/Cas9 target sites in *BmFoxO* and *BmEcR* genes. The BmFoxO and BmEcR sequences of WT are shown at the top. The target sequence of sgRNA is indicated in blue. Deletions are indicated by dashes. The mutated bases are shown in red. (C) The nucleotide sequence (NM_001202535) and deduced amino acid sequence (NP_001189464.1) of the *BmFoxO* gene. The Forkhead domain is shown in yellow background. The FOXO-TAD domain is shown in cyan background.
